# Can the mini-sling become the golden standard for treating stress urinary incontinence? Comment: The TFS retropubic tensioned minisling for SUI—a 14 year experience with high long-term RCT cure

**DOI:** 10.1007/s00192-021-04922-8

**Published:** 2021-07-21

**Authors:** H. Inoue, Y. Sekiguchi, R. Nakamura

**Affiliations:** 1 Department of Obstetrics and Gynecology, Shonan Kamakura General Hospital, Kamakura, Kanagawa Japan; 2 LUNA Pelvic Floor Total Support Clinic, Women’s Clinic LUNA Group, Yokohama, Japan

Dear Editor

We read the recent article by Cheng-Yu Long et al. with interest [1]. The data since publication of the first midurethral minisling in 2005 by Petros and Richardson [2] support the editorial statement [1]. The first midurethral minisling was, and remains, a tensioned retropubic sling. Since then, it has been validated by a 5-year RCT by Sivaslioglu who compared the TFS minisling with a TOT (transobturator tape) [3] with objective cure rates of 75% and 83% TFS at 5 years. There was one TOT erosion (2.5%) and one TFS anchor displacement in the left side. The anchor was removed under local anesthesia, and the patient remained continent.

Since 2006, our Japanese group has performed > 500 TFS minisling operations for stress urinary incontinence (SUI). We reported 90% 3-year SUI cure for TFS minisling with no erosions [4]. Five patients needed indwelling catheters, and all five patients voided without difficulty within 2 days. There were no intraoperative complications and no erosions within the 3 years. We also reported 90.9% cure at 12 months for women with intrinsic sphincter defect (ISD) (patients with maximum urethral closure pressure < 20) [5]. There was one intraoperative bladder perforation, but no erosions. All our operations were performed under local anesthetic (LA)/sedation with same-day discharge.

All operations were performed with a third-generation non-stretch lightweight tape. The TFS minisling is unique in that it is retropubic and uses a one-way tensioned tape. As such, it can be tightened millimeter by millimeter to obtain the precise tension required for closure with minimal postoperative urinary retention, an important consideration for ISD [5]. The minimal nature of the operation allows it to be done under LA. Furthermore, reports of a retropubic being superior to TOT for repeat surgery give this method an added advantage over the TOT minislings.
